# Mouse Mutants for the Nicotinic Acetylcholine Receptor ß2 Subunit Display Changes in Cell Adhesion and Neurodegeneration Response Genes

**DOI:** 10.1371/journal.pone.0018626

**Published:** 2011-04-25

**Authors:** Carol M. Rubin, Deborah A. van der List, Jose M. Ballesteros, Andrey V. Goloshchapov, Leo M. Chalupa, Barbara Chapman

**Affiliations:** 1 Department of Neurobiology, Physiology, and Behavior, University of California Davis, Davis, California, United States of America; 2 Vice President for Research, George Washington University, Washington, D.C., United States of America; 3 Center for Neuroscience, University of California Davis, Davis, California, United States of America; University of North Dakota, United States of America

## Abstract

Mice lacking expression of the ß2 subunit of the neuronal nicotinic acetylcholine receptor (CHRNB2) display abnormal retinal waves and a dispersed projection of retinal ganglion cell (RGC) axons to their dorsal lateral geniculate nuclei (dLGNs). Transcriptomes of LGN tissue from two independently generated *Chrnb2−/−* mutants and from wildtype mice were obtained at postnatal day 4 (P4), during the normal period of segregation of eye-specific afferents to the LGN. Microarray analysis reveals reduced expression of genes located on the cell membrane or in extracellular space, and of genes active in cell adhesion and calcium signaling. In particular, mRNA for cadherin 1 (Cdh1), a known axon growth regulator, is reduced to nearly undetectable levels in the LGN of P4 mutant mice and Lypd2 mRNA is similarly suppressed. Similar analysis of retinal tissue shows increased expression of crumbs 1 (Crb1) and chemokine (C-C motif) ligand 21 (Ccl21) mRNAs in *Chrnb2−/−* mutant animals. Mutations in these genes are associated with retinal neuronal degeneration. The retinas of *Chrnb2−/−* mutants are normal in appearance, but the increased expression of these genes may also be involved in the abnormal projection patterns of RGC to the LGN. These data may provide the tools to distinguish the interplay between neural activity and molecular expression. Finally, comparison of the transcriptomes of the two different *Chrnb2−/−* mutant strains reveals the effects of genetic background upon gene expression.

## Introduction

Mutant mice provide an invaluable tool for studying the development and organization of the mammalian visual system [1]. Eye specific segregation develops in the lateral geniculate nucleus (LGN) and superior colliculus of mice as an initially intermingled pattern of retinal ganglion cell (RGC) projections that gives way to eye specific regions by postnatal day 8 (P8). Mice with a deletion of the gene for the ß2 subunit of the nicotinic acetylcholine receptor (*Chrnb2−/−* mutants) have served as a popular model for studying visual system development [2], [3], [4], [5], [6], [7], [8]. In *Chrnb2−/−* mutants the projections of the two eyes remain intermingled in the LGN at P8 but form an altered eye specific segregated pattern by P14 [2], [6], [7]. Retinotopic organization is less precise [5], [8], [9], and the receptive field properties of LGN and visual cortical neurons are abnormal in the *Chrnb2−/−* mutants compared to wild type (WT) animals [3], [4], [9].

Coordinated firing of action potentials that sweep across the retina in a wavelike manner (retinal waves) occur in the WT retina from the late embryonic stage to eye opening in the mouse [2]. Retinal waves are believed to drive development of the eye specific segregation pattern in the LGN, as blocking retinal waves with intraocular injections of epibatidine blocks eye specific segregation [10], [11], [12], [13]. Application of antagonists to retinal ß2 nAChRs also blocks expression of retinal waves [14]. As expected from these results, *Chrn2b*−/− mutant mice were intitally reported to lack retinal waves [14], [15] It has recently been demonstrated that the *Chrnb2−/−* mutants do manifest retinal waves, though the waves are not normal in their spatial or temporal characteristics [15], [16].

The aberrations in the structural and functional organization of the *Chrnb2−/*− mutant animals are thought to reflect the abnormal retinal activity that occurs during the developmental period when key features of the visual system are being established. Whether or not the structural and functional abnormalities that have been documented in the *Chrnb2−/−* mutants are driven by abnormal patterns of retinal activity during development, the aberrations displayed by the visual system of the *Chrnb2−/−* mutants presumably also reflect the abnormal expression of molecules that play a role in forming the patterns of connections in the developing visual system. As a first step in probing this matter, in the present study we have used microarray technology to compare the expression of molecules in the retina and the LGN of the *Chrnb2−/−*mutants with those of WT animals during the period of eye specific segregation in the LGN.

## Methods

### Animals

All experiments were performed in accordance with NIH and institutional guidelines regarding animal use and were approved by the campus animal use and care committee of the University of California, Davis. WT (C57BL/6J) mice were obtained from Jackson Laboratory (Bar Harbor, Maine), Picciotto (“Pic”) *Chrnb2−/−* mutant mice were a kind gift from Dr. M Picciotto [17] and Xu mutants were derived from embryos (ES Cell line ID 00211-UNC) supplied through the Mutant Mouse Regional Resource Center (University of California, Davis, California). Neonatal mice were administered a lethal IP dose (0.05–0.1 ml) of Fatal Plus (Vortech Pharmaceuticals; Dearborn, MI) at the time of tissue collection. Tail snips were collected from mutant mice for genotyping to confirm mutation.

### Microarray tissue preparation, hybridization, and analysis

Total retinas from three male P4 littermates from timed pregnancies were harvested and immediately placed on dry ice. Tissue was maintained at −80°C until RNA was prepared. Mice were homozygous Picciotto *Chrnb2−/−*, Xu *Chrnb2−/−*, or C57BL/6J WT animals. Retinas from each animal were separated from the sclera and were combined. RNA was isolated with the RNAeasy Mini kit (Qiagen). A total of 500 ng of RNA was amplified with Ambion MessageAmp II-Biotin Enhanced reagents (AM1791) and aRNA yields were 57–122 µg. Twenty micrograms of each aRNA target (9 samples total) were fragmented and hybridized to Affymetrix GeneChip Mouse 430 2.0 expression arrays using the Affymetrix GeneChip Fluidics Station and Affymetrix reagents (Affymetrix). Adult retinal tissue was prepared as above but only two male littermates of each type were sampled.

Total LGN (including both dorsal and ventral portions) was isolated from a second set of male P4 littermates under the dissecting microscope. The cortex was removed and the LGNs were visualized and excised using an 18G needle. The LGN was isolated as a discrete entity without any surrounding thalamic tissue attached. Left and right LGN were combined for each animal and frozen on dry ice. RNA was prepared and amplified as described for retinas, but the aRNA yield from 500 ng of LGN RNA was much lower, 10–31 µg. Ten micrograms of each aRNA target (9 samples total) were fragmented and hybridized as described for retinal samples.

Both the retinal and LGN tissue contain a mixture of cell types.

Chip data were analyzed with dChip software [18], using quantile normalization and PM/MM modeling. Present calls were above 50% for all samples (Table S1). Thresholds chosen for WT vs. mutant analyses were 1.5-fold minimum change between WT and KO and difference ≥50, and probe sets in both Picciotto and Xu mutants had to differ from WT in the same direction (i.e., either overexpressed in WT or overexpressed in both mutants). The difference parameter excludes probe sets detected only at very low levels. For Picciotto vs Xu mutant analyses the minimum fold change was increased to 2.0. One P4 LGN WT hybridization (WT E) showed a high percentage of outliers and was omitted from analysis (Table S1). Excel data files using the threshold values described above are presented as Tables S2 and S3. Affymetrix CEL files are available under GEO Series Record #GSE22824.

### Immunohistochemistry

Following euthanasia, eyes from WT and mutant animals were enucleated then fixed in 4% PFA in PBS for 30–45 minutes. After cryoprotection in a 25% sucrose solution each eye was embedded in OCT (Ted Pella, Torrence, CA), sectioned at 10 µm on a Leica (Deerfield, IL) cryostat, and mounted on glass slides. Brains from WT and mutant animals were harvested and the unfixed brain was embedded in OCT, frozen on dry ice, sectioned at 15 µm on a cryostat (Leica), mounted on glass slides and stored at −80°C until used. Before processing for immunohistochemistry, brain section slides were warmed to room temperature, fixed in 4% PFA for 2–4 minutes and washed in PBS.

For immunostaining, sections were blocked in 10% normal donkey serum, 2% bovine serum albumin, and 0.3% Triton X in PBS for 2 hours, then incubated in blocking solution overnight at 4°C with primary antibodies used as follows: Anti-CHRNB2 M270 (1∶250 n8408, Sigma, St Louis, MO), anti-CHRNB2 (1∶250, sc-1449, Santa Cruz Biotechnology, Santa Cruz, CA), anti-CCL21 (1∶500; sc-25445, Santa Cruz Biotechnology), anti-CDH1 (1∶50; 610181,BD Biosciences, San Jose CA) and anti-SPP1 (1∶100; 01-20002, American Research Products, Belmont MA), followed by incubation with Alexa Fluor 568 or 594 fluorescent secondaries (1∶500; Invitrogen, Carlsbad, CA), or CY3 (1∶500; Jackson ImmunoResearch Laboratory., Inc., West Grove, PA) for 1 hour. Nuclei were visualized with DAPI (1∶500; KPL, Gaithersburg, MD) and sections were coverslipped using Vectashield (Vector Laboratories, Burlingame, CA). For control slides primary antibodies were omitted. Images were acquired on an Olympus FV500 confocal microscope (Olympus, Japan). Brightness and contrast were adjusted using Adobe Photoshop.

### Retinal projections to LGN

Retinal projections were traced by making intraocular injections of cholera toxin-ß (CTB) conjugated to fluorescent dyes (Molecular Probes). Twenty four hours prior to the required time points, (P4, P8) mice were anesthetized by immersion in ice water and intraocular injections of 1 µl of CTB conjugated to Alexa 488 (left eye) and Alexa 594 (right eye) in 0.5% saline were made into the far temporal region of the eye with a micro pipette. The eyes were treated with ophthalmic antibiotic and the animals placed on a warm surface until full movement was regained. After euthanasia, the brains were removed and immersion fixed with 4% paraformaldehyde for 48 hours. The brains were then sectioned at 50 µm on a vibratome (Leica), mounted on glass slides, cover slipped, then imaged on a Nikon Eclipse E600 upright microscope equipped with an ORCA-ER C4742-90 CCD camera (Hamamatsu Photonics) using a 10× objective. Images were pseudo colored using Wasabi software (Hamamatsu).

### RT-PCR

Qualitative RT-PCR was performed to validate the microarray assay for selected genes of interest. Qiagen OneStep RT-PCR reagents were used with the protocol recommended by the manufacturer. Ten to 20 ng of each template RNA was used per 25 µl reaction volume. Negative controls were run without added template. At 20–35 cycles aliquots of each reaction were withdrawn and run on agarose gels. Gel images have been adjusted for brightness and contrast with Adobe Photoshop. Actin control RT-PCRs are shown in Figure S1. Primers used are given in Table 1.

**Table 1:** RT-PCR primers.

Gene symbol	Tissue ampli-fied	Forward primer	Reverse primer	Expected cDNA size (bp)	Comment
Actb	Retina, LGN	gaaatcgtgcgtgacatca	aacgcagctcagtaacagt	535	
Chrnb2	Retina, LGN	gtatcattggcacagctca	gcaatgaagcgtacaccgt	1120	
Pisd-ps3 (Rik4933439C20)	Retina, LGN	ctcttggtggtctttcaag	agaaactctacagacgcca	270	Multiple chromosomal loci
Plac9	Retina	aggcgactacggacaaact	ttgcacaggtcacccaggt	590	
Ccl21 (6CKine)	Retina	gatgactctgagcctcctt	gtctgttcagttctcttgca	370	
Rik4933409K07	Retina	caagtctgtgttgacatgga	ttaataatgtacagcagagaca	715	
Crb1	retina	cttgtgtctgccctcaaga	gtgcagcccaggagaattt	710	4 known isoforms
Xlr3a	LGN	caattactggttagcacacat	tatccatacaagtgagggat	420	
Spp1	LGN	agaagcatccttgcttgggt	cttcatgtgagaggtgaggt	615	
Cdh1	LGN	cccaagttgcccagttct	atcttagagaacggtttcaat	545	
Lypd2	LGN	ttggcactgatattgggca	ccatggctttacagcagga	370	

## Results

### Genetic background of the two *Chrnb2−/−* mutant strains contributes to changes in their transcriptomes

**Figure 1:**
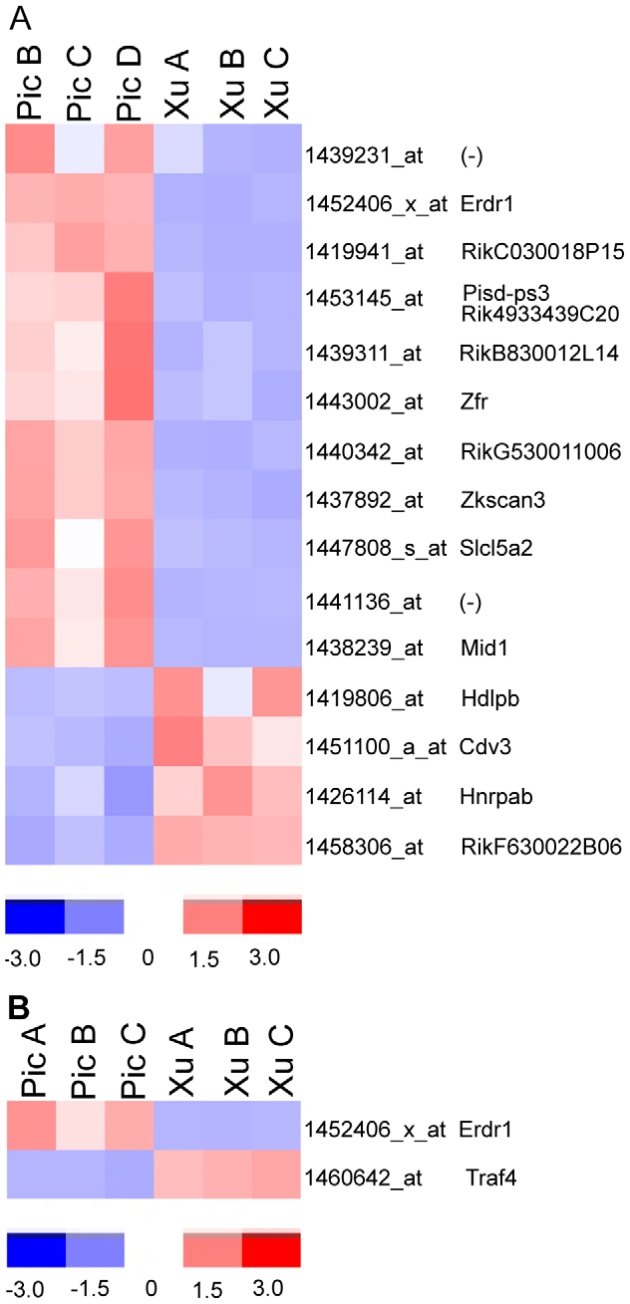
Differential gene expression between P4 Pic and Xu Chrnb2−/− mutant retinas and LGNs. A. Heat map of genes differentially expressed between the two mutant P4 LGNs. B. Heat map of genes differentially expressed between the two mutant P4 retinas. Gene names are in Table S4.

Comparison of the two mutant strains with each other and with the WT strain (C57BL/6J) reveals the genes truly affected by lack of *Chrnb2* expression *vs.* those resulting from inter-strain differences. The importance of the contribution of the mouse background strain to the transcriptional profile has been noted by others [19], [20], [21], [22]. When gene expression is compared in Xu and Picciotto P4 LGN, 15 genes display significantly altered expression by the chosen stringency criteria (Figure 1A). Two genes (*Erdr1* and *Traf4*) differ between the two mutants in the P4 retina (Figure 1B), while hundreds of genes showed different expression between the two mutant adult retinas (Table S3). However, the difference in *Erdr1* and *Traf4* expression is no longer evident in the adult retina. Only two adult retinas were sampled for each type of mouse, vs. three each for P4 mice, and many lens proteins appear in the differentially expressed genes in the adult mice. We attribute most of the variation in the adult samples to the small sample size and dissection artifacts.

Expression of at least two genes which show significantly different expression between mutant and WT animals may also be affected by background strain. The *Xlr3a* gene, which is overexpressed in P4 mutant LGN (see below), is located on the X chromosome. The two *Chrnb2−/−*mutant strains potentially express combinations of X alleles from the 129/SvEv, HM1, DBA/2 and/or C57BL/6J lines [20]. Increased expression of this gene in mutant LGNs may reflect the differences in X chromosome alleles relative to C57BL/6J control expression rather than lack of CHRNB2. As previously mentioned, expression of the *Pisd-ps3* pseudogene family is reduced in mutants for both tissues and ages examined. However, two probe sets (*1453144_at* and *1453145_at*) are also detected significantly less in Picciotto than in Xu *Chrnb2−/−*mutant mice, indicating a potential background strain effect for at least some of these loci.

### *Chrnb2* mRNA expression is downregulated in P4 mutant mouse tissues

**Figure 2 pone-0018626-g002:**
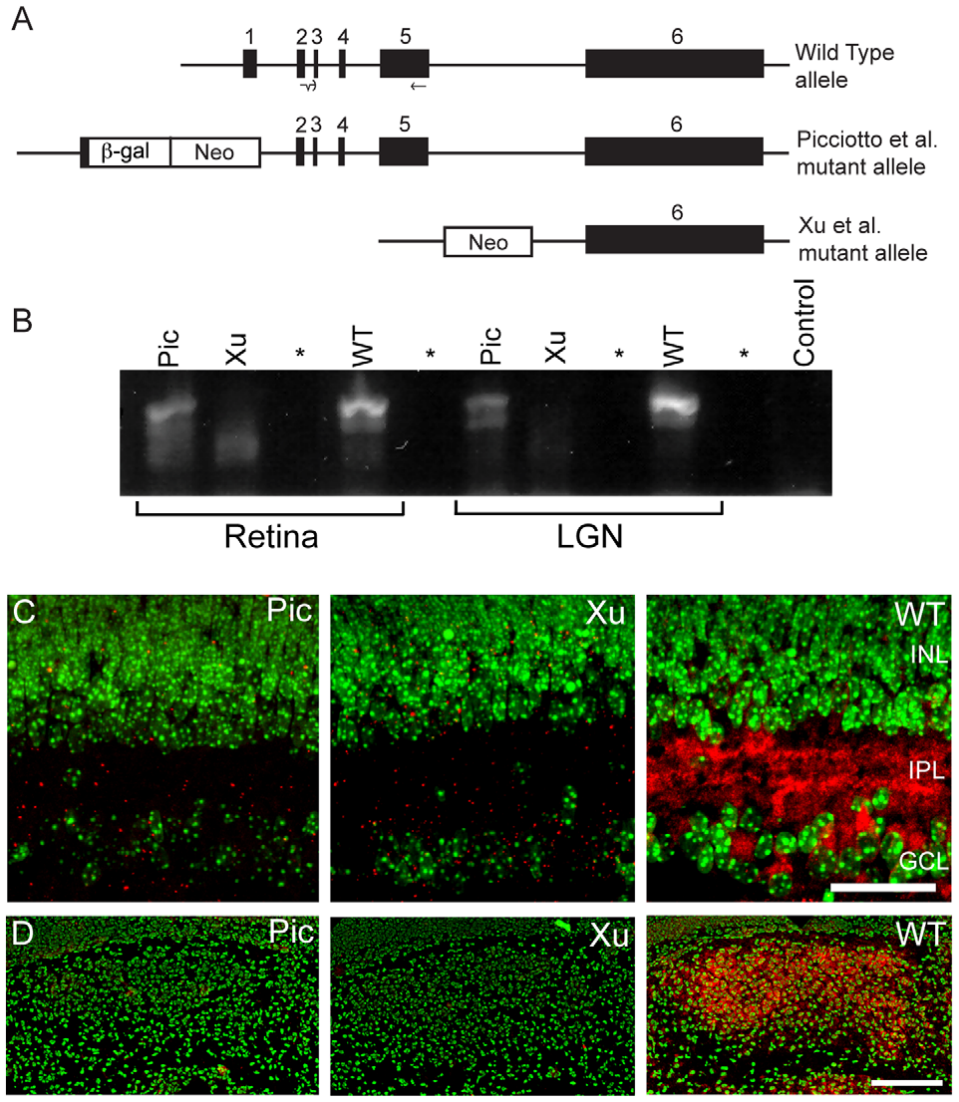
Genotypes and validation of Chrnb2−/− mutants. A. The structures of the Chrnb2 gene in WT and Pic (Piccotto) and Xu mutants. Exons are numbered solid boxes. The positions of the primers used in (B) are solid black lines and arrows shown below the WT diagram. B. RT-PCR amplifications of Chrnb2 from P4 retinal and LGN mRNA. The forward primer spans the exon 2–3 junction. The reverse primer is located at the 3′ end of exon 5. (see A) Primer sequences are given in Table 1. The expected cDNA product is 1120 bp. Note that Pic mice express some Chrnb2 mRNA while Xu mice do not. This image was made of a 35-cycle amplification to visualize expression in the Pic mice clearly. At 25 cycles the band in the Pic lanes is barely visible (not shown). * indicates an unloaded lane. “Control” is without template. Actin amplification controls are shown in Figure S1. C. Confocal images of retinal tissue using an antibody to CHRNB2 (sc-1449). INL; inner nuclear layer; IPL, inner plexiform layer; GCL; ganglion cell layer. Scale 50 µm. D. Confocal images of P4 LGN tissue using an antibody to CHRNB2 (sc-1449). The region occupied by the LGN expresses CHRNB2. Scale 200 µm. In C and D, DAPI counterstain is pseudocolored green and anti-CHRNB2 is red.

The Picciotto and Xu *Chrnb2−/−*mutants are created by two distinct procedures. In the Picciotto mutant a small portion of the gene around the start codon and the signal peptide has been replaced with a lacZ-neomycin resistance construct [17]. Xu mutants have the first five (of six total) exons replaced with a neomycin resistance cassette [23] (Figure 2A). Expression of *Chrnb2* mRNA was dramatically lower in microarray analysis of tissues from both mutants (Table 2). However, the *Chrnb2* probe set *1436428_at* consistently showed less expression in Xu animals than in Picciotto animals (Table 2). To determine whether Picciotto mice make a small amount of *Chrnb2* mRNA, RT-PCR was performed with primers spanning exons 3–5 (Figure 2A and B). Several in-frame potential start codons immediately downstream of the canonical AUG start codon remain in Picciotto mice and *Chrnb2* mRNA is detectable in Picciotto P4 retina and LGN, while no *Chrnb2* mRNA is detected in Xu mutant amplifications (Figure 2B). The amount of mRNA made is small, and CHRNB2 protein is not detected in Picciotto retina by Western blot [17] or by IHC (Figure 2C and D). Furthermore, the phenotypes of the Picciotto and Xu animals with respect to retinal wave activity and LGN segregation are similar [15], suggesting that the small amount of mRNA made in Picciotto animals does not affect their mutant status. However, detection of this mRNA on the microarray scan and its subsequent verification by RT-PCR analysis indicates the sensitivity of the microarray assay.

**Table 2:** Chrnb2 mRNA expression in P4 Xu and Picciotto Chrnb2−/− LGN and retina vs WT.

LGN expression	probe set	WT A	WT D	*	KO A	KO B	KO C	fold change
Xu vs WT	1436428_at	313	296		8	5	4	60.4
Xu vs WT	1441837_at	86	85		8	12	16	7.5
Pic vs WT	1436428_at	313	296		39	57	54	6.0
Pic vs WT	1441837_at	86	85		12	12	8	8.0

Retina expression		WT A	WT B	WT C	KO A	KO B	KO C	fold change
Xu vs WT	1436428_at	103	127	124	19	9	14	8.9
Xu vs WT	1441837_at	12	8	18	30	9	8	0.81
Pic vs WT	1436428_at	103	127	124	26	30	25	4.4
Pic vs WT	1441837_at	12	8	18	11	11	13	1.1

*Only two WT LGN microarrays were used (see Methods).

### Expression of calcium signaling and cell adhesion genes is reduced in P4 mutant LGNs

**Figure 3 pone-0018626-g003:**
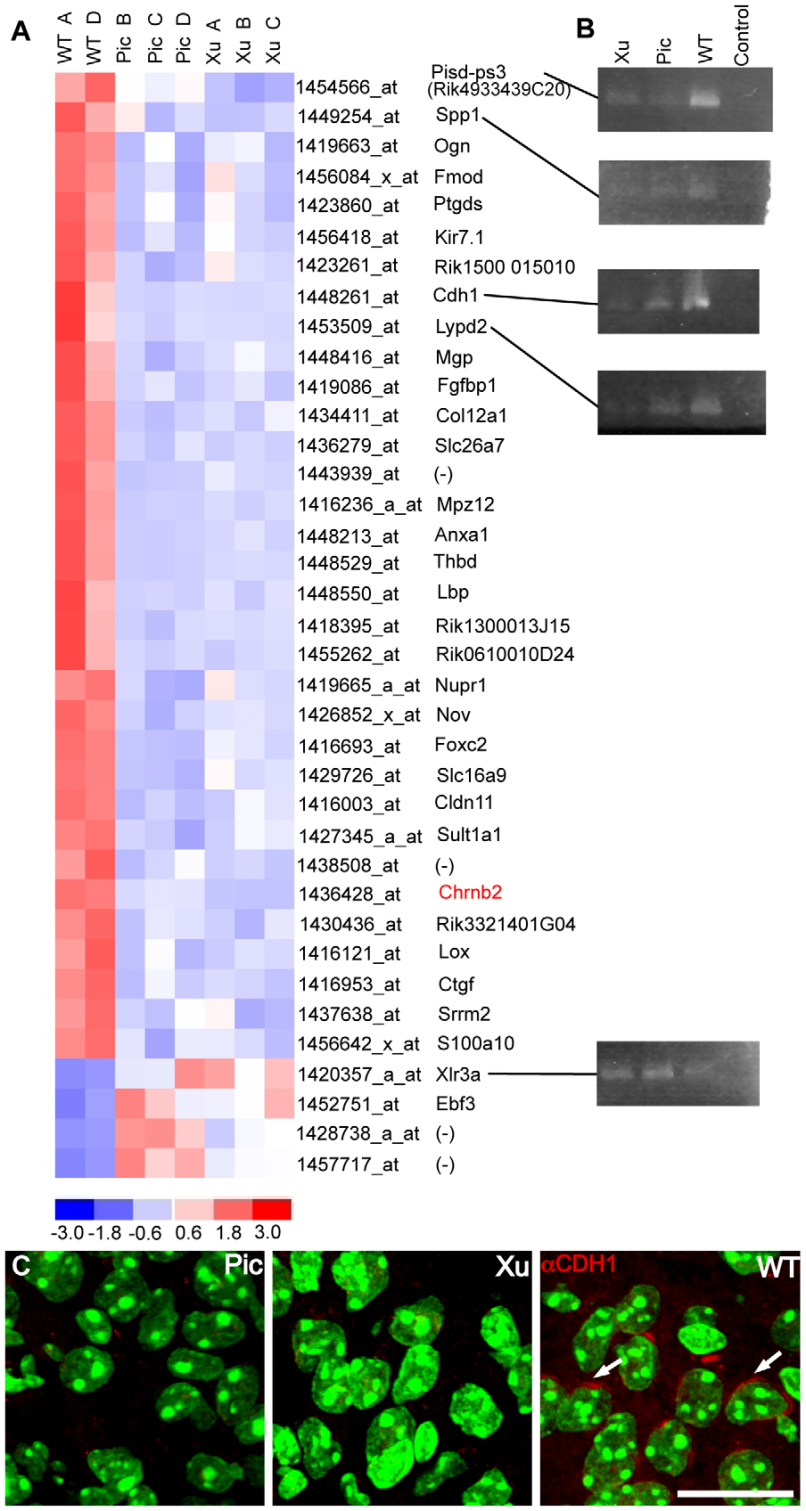
Differential CDH1 expression in P4 LGN. A. Heat map of genes differentially expressed between both mutants and WT selected from the microarray assay. Note that only four genes are overexpressed in mutant LGN and expression of these differs greatly between the Pic and Xu mutants. Redundant probesets have been stripped from the map. When a gene symbol is available it appears next to the probeset. Gene names are in Table S4. B. Validation qualitative RT-PCRs for some of the genes in (A). “Control” is without template. Primers used are given in Table 1. Actin amplification controls are shown in Figure S1. C. Confocal images of tissue sections demonstrating the absence of CDH1 protein in mutant LGN. Nuclei are counterstained with DAPI and pseudocolored green. CDH1 (arrows) is expressed discretely within the cell membrane. Scale bar 25 µm.

Exclusive of the *Chrnb2* index gene, 32 transcripts are downregulated in mutant P4 LGNs using the selected stringency criteria (Figure 3A;Table S2). Of the annotated genes, 22 are membrane proteins or are found in the extracellular matrix. Of special interest among these is Lypd2 (Lynx2), a known endogenous bungarotoxin-like inhibitor of nAChRs. Five transcripts are associated with calcium binding and/or signaling (*Anxa1*, *Cdh1*, *Mgp*, *S100a10* and *Thbd*) and six have known cell adhesion functions (*Cdh1*, *Cldn11*, *Col12a1*, *Ctgf*, *Mpzl2*, and *Spp1*). *Cdh1* (E-cadherin, cadherin 1) expression is remarkably suppressed: (12 to 18 fold less in both mutant strains vs WT, Figure 3A; Table S2) and this difference in expression was confirmed by RT-PCR and IHC (Figure 3B and C).

Only four transcripts are upregulated in P4 *Chrnb2−/−*mutant LGN by the criteria chosen (Figure 3A). These genes show considerable variability between the two types of mutant and may be differentially expressed due to the different genetic background of the three types of mice compared (see above).

**Figure 4:**
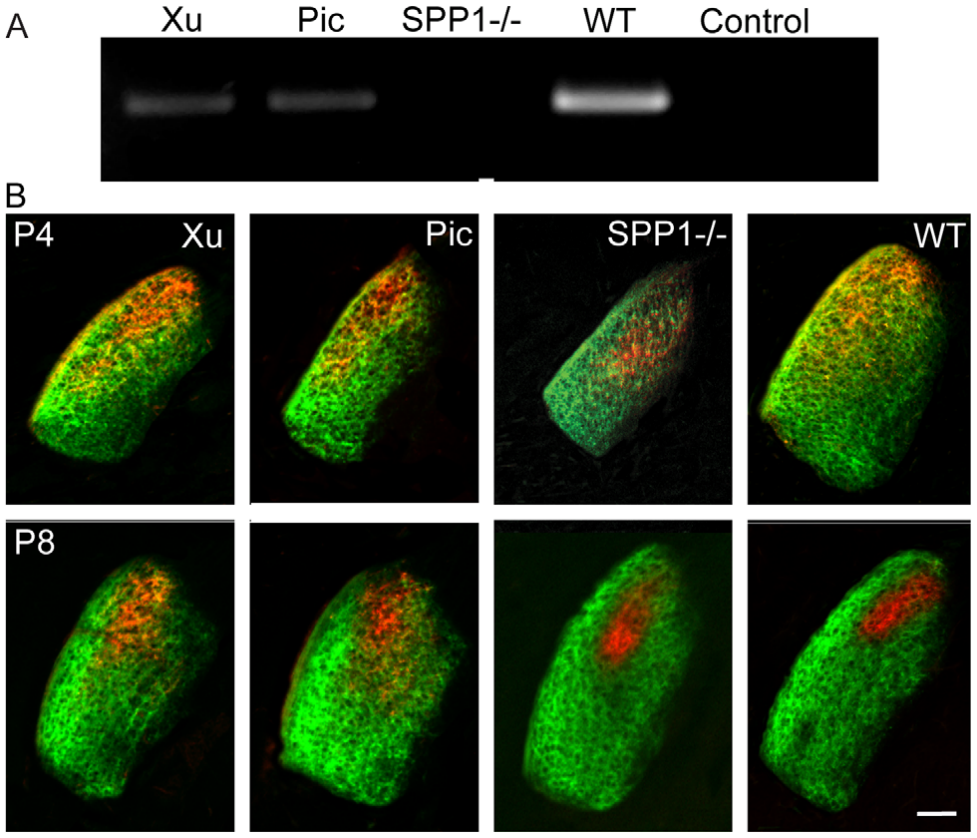
Spp1 expression and RGC segregation in WT Spp1−/− and Chrnb2−/− LGN. A. Validation qualitative RT-PCRs for the Spp1 gene in P4 WT, *Chrnb2−/−* and *Spp1−/−* LGNs. Spp1 RNA amplification is low in *Chrnb2−/−* LGN and is not evident in *Spp1−/−* LGN compared to WT. Actin amplification controls are shown in Figure S1. B. RGC segregation in WT and mutant LGN. At P4 eye specific segregation has not developed in any animals tested. At P8 eye specific segregation in the LGN has developed in WT and *Spp1−/−* mutants but not in *Chrnb2−/−* mutants. Choleratoxin-ß conjugated to Alexa 594 (red) and Alexa 488 (green); Scale 100 µm.

SPP1 is a known axon guidance regulator [24]. *Spp1* mRNA was confirmed to be reduced in P4 *Chrnb2−/−*mutant LGN and absent from *Spp1−/−* P4 mutant LGN by RT-PCR (Figure 4A). At P4, LGN eye specific segregation is not evident in WT, *Chrnb2−/−* or *Spp1−/−* mutants. At P8, eye specific segregation has developed in the WT and *Spp1−/−* mutant but not in the *Chrnb2−/−*mutants (Figure 4B).

### Expression of genes associated with neuronal degradation is increased in P4 but not in adult *Chrnb2−/−* mutant mouse retinas

**Figure 5:**
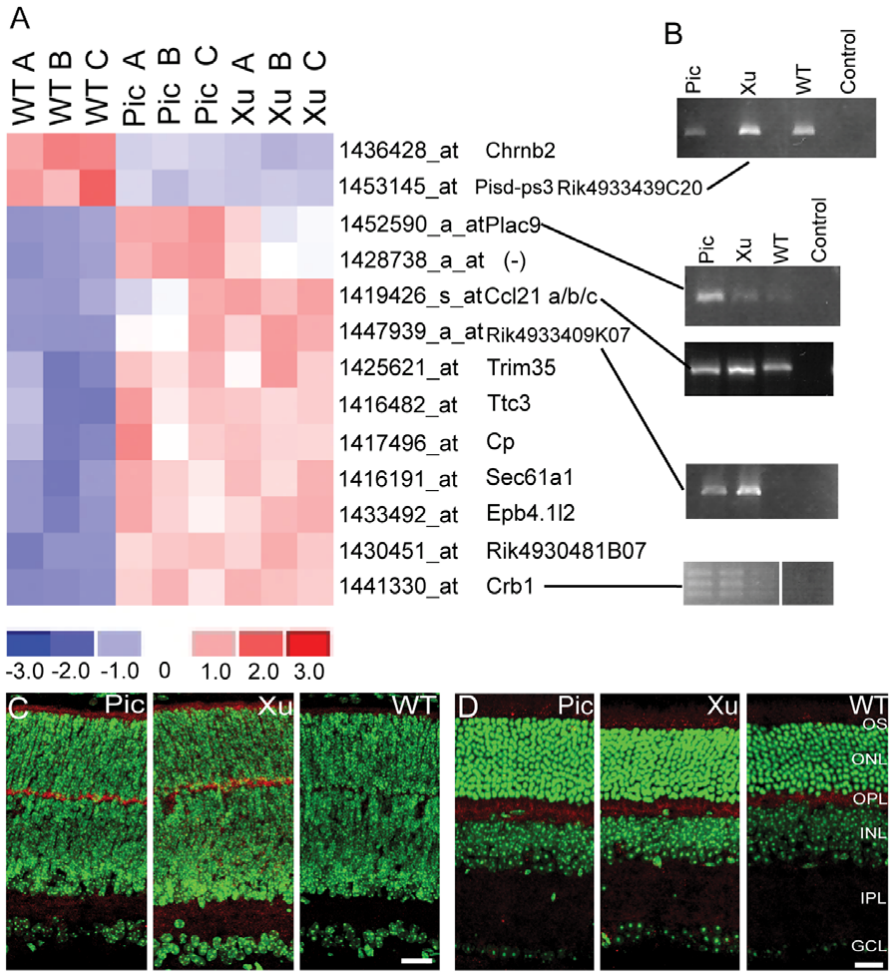
Differential gene expression in P4 retina. A. Heat map of genes differentially expressed between both mutants and WT selected from the microarray assay. Note that only one gene other than Chrnb2 is downregulated in the mutants (Pisd-ps3). Redundant probesets have been stripped from the map. When a gene symbol is available it appears next to the probeset. Gene names are in Table S4. B. Validation qualitative RT-PCRs for some of the genes in (A). “Control” is without template. Primers used are given in Table 1. Amplification with Crb1 primers yields three bands. The Expasy (www.expasy.org) annotation for mouse Crb1 gives four known splice variants. C. Confocal images of P4 retina using an antibody to CCL21 (aka 6CKine). CCL21 protein is strongly expressed in the outer segment (OS), and outer plexiform layers (OPL) and weakly in the inner plexiform layer (IPL) of *Chrnb2−/−* but not WT retinas. D. Confocal images of adult retina using an antibody to CCL21 (aka 6CKine). CCL21 protein appears in the outer segment (OS) and outer plexiform layer (OPL) of all animals. Anti-CCL21 (red), DAPI nuclear counterstain (pseudo colored green). Scale 25 µm.

In contrast to the P4 LGN in which nearly all differentially expressed genes showed decreased expression in mutants, 11 of 12 genes (*Chrnb2* excluded) with significantly altered expression in P4 retina show an increased expression in the *Chrnb2−/−*mutants (Figure 5A; Table S2). Seven of these gene products are found in the membrane and extracellular space (EPB4.1, EPB4.1L2, D14ERTD449E, SEC61A1, CRB1, CCL21 and CP), as is the index protein CHRNB2. Three products of upregulated genes (CRB1, CP and CCL21) are associated with retinal injury or degenerative processes. Although expression of these genes is upregulated in P4 *Chrnb2−/−*mutant mice, retinas from these mice are phenotypically normal (Figure 2C, 5C and D). Immunohistochemistry confirmed the presence of CCL21 protein in *Chrnb2−/−*mutant retina outer segment (OS) and outer and inner plexiform layers at P4 compared to WT (Figure 5C). Microarray comparisons of adult retinas showed little consistency with expression changes found in P4 retina. Of particular interest, elevated CCL21 protein levels were detected by IHC in *Chrnb2−/−*mutant retinas at P4, as predicted by the microarray result, but not in adult mutant or WT retina, also consistent with the microarray result (Figure 5D).

### Effects of the *Chrnb2−/−* mutation are tissue- and temporally specific

**Figure 6:**
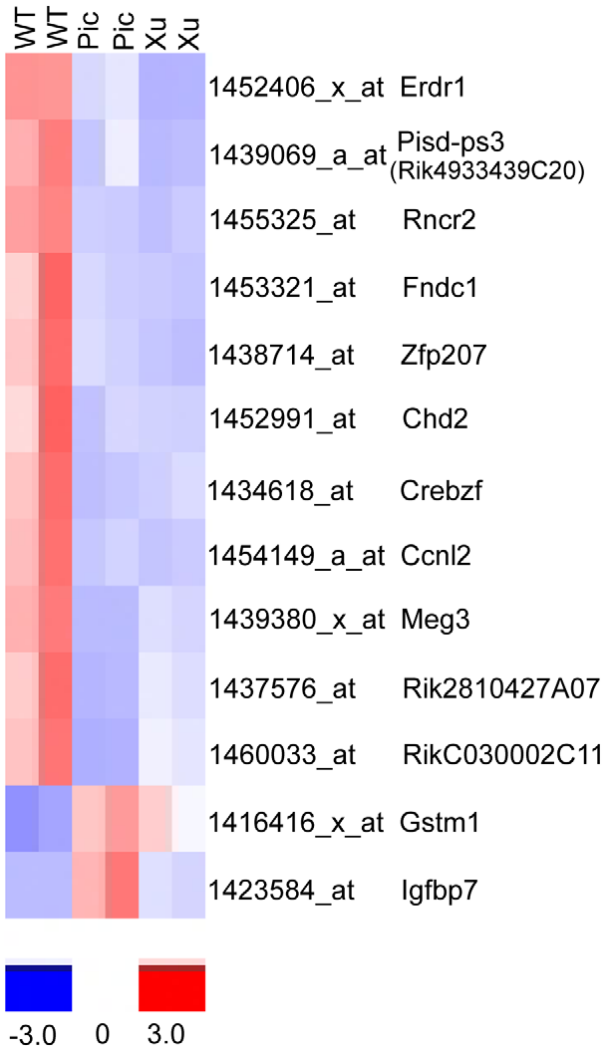
Differential gene expression in adult retina. Heat map of genes differentially expressed between both mutants and WT selected from the microarray assay. Redundant probesets have been stripped from the map. When a gene symbol is available it appears next to the probeset. Chrnb2 does not appear because expression levels lie below the chosen threshold levels for both probesets in all retinas.

Only one set of microarray probes, for *Pisd-ps3 (Riken 4933439C20*) shows reduced expression in both mutants in P4 LGN and in P4 and adult retinas by the selection criteria chosen (Figures 3A, 5A, and 6). The microarray probes detect transcripts of this phosphatidylserine decarboxylase pseudogene that arise from loci on five different chromosomes (5, 11, 14, 17 and Y). While expression is significantly reduced in both types of mutant P4 LGNs vs WT, Xu mutants show considerably less expression than Picciotto mutants for at least three probe sets (Table S2), raising the possibility that expression is affected by genetic background in addition to the *Chrnb2−/−* mutation.

Expression of cdh1 mRNA is low and indistinguishable in both P4 and adult mutant and WT retinas, but expression of CDH1 is considerably reduced in P4 *Chrnb2−/−*mutant LGN (Figure 3C). Lack of CDH1 is an embryonic-lethal defect in mice [25], and both *Chrnb2−/−*mutants develop normally to adulthood. Either enough CDH1 is made in *Chrnb2−/−*mutants to assure viability (though below the detection threshold of IHC), or the reduced expression in P4 LGN is localized. Expression of the immune response indicator CCL21 (chemokine (C-C motif) ligand 21; 6CKINE) is increased in P4 *Chrnb2−/−*mutant retina, but not in adult mutant retina (Figure 5C and D). Microarray analysis reveals no difference in Ccl21 mRNA expression between mutant and WT P4 LGN.

## Discussion

### *Chrnb2−/−*mutant mice display altered retinal waves and abnormal neuronal connections in the visual circuitry of the LGN

Mice lacking the Chrnb2 subunit exhibit abnormalities in their visual circuitry. Retinal ganglion cells of WT mice display retinal waves before eye opening, but *Chrnb2−/−*mutants have been reported to lack retinal waves between P1–P8 and lack refinement of projecting RGCs within the LGN [2]. It has been proposed that spontaneous retinal wave activity is a prerequisite for correct eye-specific axonal segregation in the LGN [7], [26], and the findings that *Chrnb2−/−*mutant mice lacked both cholinergic retinal waves and refined axonal targeting to the LGN supported this hypothesis.

Recently, this laboratory has reported that two strains of *Chrnb2−/−* mutant mice [17], [23] do display spontaneous retinal waves at P4–P5, but the waves are not normal in their spatio-temporal patterning. We also confirmed that RGC targeting abnormalities are present in the LGN for both *Chrnb2−/−* mutants despite the presence of spontaneous retinal waves [15]. These results indicate that the presence of these abnormal retinal waves is not sufficient to drive development of a normal pattern of eye specific segregation in the LGN [15], [16]. It is not clear what aspects of retinal activity patterns may be involved in the normal development of retinogeniculate projections (for review see [27]). However, it seems clear that altered patterns of gene expression must be involved in the altered RGC projection pattern seen in the mutant animals. To determine what molecular factors might be associated with *Chrnb2* mutation at the age when cholinergic waves are normally present (P1–P8), we assayed RNA populations in both mutant and WT (C57BL/6J) mouse LGN and retinal tissue at P4 by microarray hybridizations. Adult retinas were also assayed and gene expression compared to age matched WT animals.

### Comparison of two different mutants relative to wild type controls reveals the effect of background strains upon the RNA population

While the use of single-gene mutants to elucidate gene function is a powerful tool, many authors have cautioned that the methods by which these knockout animals are prepared may not reveal the true contribution of the knocked out gene to the transcriptional profile compared with “control” animals [19], [20], [21]. The genetically “pure” C57BL/6J strain, the strain used as the control in our study and a genetic contributor to both *Chrnb2−/−* mutants is heterozygous for at least one chromosomal locus [28]. Kedmi and Orr-Urtreger [19] compared brain RNA from *Chrnb4*−/− mutants with C57BL/6J in a microarray analysis. The *Chrnb4−/−* mutants used were generated by a process similar to the Xu *Chrnb2−/−* mutants presented in this work [23]. Kedmi and Orr-Urtreger report that ten of the 77 genes with altered expression in the Chrnb4 mutants were co-localized with the *Chrnb4* gene on chromosome 9 [19]. Thus, 13% of the differences they found could result from the recombination that created the mutant, leading to altered expression of adjacent genes on the same chromosone.

*Chrnb2* is located on Chr3 and examination of our data reveals that four genes we score as having significantly different expression are also located on chromosome 3: *S100a10* and *Cldn11* in P4 LGN, *Cp* in P4 retina, and *Gstm1* in adult retina. Of these, *S100a10* and *Gstm1* lie very close to *Chrnb2* at Chr3F and differences in their expression may be a result of the mutation process rather than the lack of CHRNB2.

We find no genes differentially expressed in common with mice lacking the *Chrnb4* gene, but our tissue, ages, and selection criteria all vary from that report [19]. However, mutation of *Chrnb4* does lead to changes in expression of calcium ion binding proteins similar to our results for *Chrnb2*, indicating that this may be a pathway generally sensitive to alterations in nAChR subunit composition. While comparing both independently produced *Chrnb2−/−* animals to the selected WT strain does not completely correct for allelic effects, our analysis of two independently generated mutants allows a more certain determination of transcripts affected by the lack of CHRNB2. At least two genes with consistently different expression between both mutants and WT (*Xlr3a* and *Pisd-ps3*, see Results) appear to show an additional background strain effect on their expression.

### Genes overexpressed in P4 mutant retinas are associated with membranes and neuronal injury

In P4 retinas, mutation of *Chrnb2* is generally associated with an increase in expression of eleven transcripts and decreased expression of only one, the Pisd-ps3 family. Three genes overexpressed in P4 *Chrnb2−/−* mutant retina relative to WT are associated with retinal injury (*Crb1*, *Ccl21a/b/c*, and *Cp*). CCL21 is a ligand expressed by injured neurons that activates microglia as part of the inflammatory response. Retinas from mice lacking CHRNB2 show 8-fold overexpression of Ccl21 by microarray analysis. CCL21 is overexpressed in the OS, OPL and IPL of P4 *Chrnb2−/−* retinas compared with WT. Photic injury to the adult retina has been reported to increase expression of one member of the CCL21a/b/c chemokine family, *Ccl21a* (aka Scya21a) by more than four-fold in the ONL [29].

Why the P4 *Chrnb2−/−* mice, which have not been exposed to abnormally high light levels, synthesize large amounts of CCL21 in their retinas is not known. Unlike the pyknotic nuclei and other cellular abnormalities reported in photic injured retinas [29], gross retinal anatomy appears normal in the *Chrnb2−/−* mutant mice. Adult brains of Picciotto *Chrnb2−/−* mutants have been reported to show increased neuronal atrophy and microgliosis [30], but whether these changes are accompanied by increased CCL21 expression in aged mutant brain is not known.

Expression of ceruloplasmin (*Cp*) mRNA has also been reported to be upregulated in response to photic damage [31] and we find overexpression of *Cp* mRNA in P4 *Chrnb2−/−* mutant retina relative to WT (see Results). CP facilitates iron transport and serves as an antioxidant by oxidizing the free radical catalyst Fe^2+^ to Fe^3+^ [32]. CP has been previously reported in healthy mouse [33] and human retina [34]. Mouse and human glaucomatous retinas show an upregulation of CP during the period when RGC death is occurring due to increased intraocular pressure [35]. It is of interest to note that CP in the glaucomatous retina is localized to Müller cells in the inner nuclear layer and astrocytes in the RGC layer. We did not conduct immunohistochemistry for CP, but if increased CP is localized in the RGC layer of mutant animals, it would provide evidence that P4 *Chrnb2−/−* mice have higher rates of RGC death compared to WT animals. Although RGC death cannot directly explain the altered segregation pattern in the mutant mice, it may alter the molecular gradients or retinal activity levels in the *Chrnb2−/−* mutant retina that lead to this developmental aberration.

Mutation of CRB1 (crumbs1) is associated with Leber's congenital retinopathy and retinitis pigmentosa in humans [36], [37], [38] We see high *Crb1* expression in P4 *Chrnb2−/−* retinas compared to WT animals. CRB1 is localized in the subapical region of Műller glial cells in the mouse retina [38], a structure adjacent to the adherens junction. In P4 retina expression of the adherens junction component CDH1 is low and similar in WT and mutant mice, but expression of CDH1 is reduced in LGNs of *Chrnb2−/−* mice at this age. Xu et al [39] examined WT retinas at E12, E14, E16, P7, P14 and P30. *Cdh1* mRNA was transiently detected only at P14, in a subset of RGCs. It is possible that transient *Cdh1* expression at an age they did not assay (e.g., E17 – P4) may be eliminated by lack of CHRNB2 in the retina, and that this transient defect in the adherens junction leads to subsequent compensatory overexpression of CRB1.

CRB1 shares calcium binding properties with CHRNB2 and activation of nicotinic AChRs increases calcium trafficking at the synapse [40], [41]. Calcium is a known neuronal transcriptional regulator [42] and a lack of the CHRNB2 subunit in retina may affect transcription by altering Ca^2+^ ion flow at the synapse. CRB1 is required to prevent light-induced retinal degradation in Drosophila [43] and mice [37]. We speculate that increases of CCL21, CP, and CRB1 may indicate the death of a subset of RGCs that contain molecular markers which determine the localization of the RGC axons in the LGN. A similar process occurs in the formation of superior colliculus topography [44]. Death of a significant number of RGCs that project to a specific region could cause the altered segregation pattern evident in *Chrnb2−/−* LGN.

### *Chrnb2−/−* mutation in P4 LGNs is associated with reduced expression of the axon growth regulators cadherin 1, Lypd2 and secreted phosphoprotein 1

Knockout mutation of *Chrnb2* is associated with a decrease in transcript expression in the P4 LGN as 32 of 36 transcripts with altered expression show a reduction of expression in the mutants (see Results). Mutant LGNs at P4 show a diffuse segregation pattern of ipsilateral and contralateral RGC axons compared with WT [6], [15]. The segregation begins to resolve with age, but never becomes as focused as in the wild type animals [6]. These findings indicate that the formation of a normal eye-specific segregated pattern may require an expression pattern of specific genes during the first postnatal week that is altered in the *Chrnb2−/−* mutant LGNs.

Given the displacement of RGC axonal projections in the *Chrnb2−/−* mutant LGNs, it is of interest that expression of mRNAs encoding SPP1 (secreted phosphoprotein 1, osteopontin), LYPD2 (LY6/PLAUR domain containing 2, Lynx2) and CDH1, proteins with known axon guidance properties, are significantly lower in P4 mutant LGN compared to the age matched WT. Various effects of SPP1 on axon growth have been reported. Ries et al [45] find a mild enhancement of axon growth by SPP1 in an *in vitro* assay using purified rat E20 RGCs while in an *in vitro* growth assay of effects on dorsal root ganglia showed SPP1 in the substrate inhibited outgrowth and caused fasciculation [24]. *Spp1−/−* mutant mice are grossly normal [46] and the RGC projection pattern in the LGNs of P4 *Spp1−/−* mutant mice appears equivalent to WT. Reduced SPP1 does not cause the segregation defect.

The association of SPP1 expression with neuronal degeneration in the rodent brain [47] and retina [48] is of interest. We find increased expression of mRNAs for two genes associated with retinal injury, *Ccl21* and *Crb1*, in *Chrnb2−/−* mutant retinas at P4 and decreased expression of *Spp1* mRNA in mutant LGNs at P4. Zoli et al [30] describe neurodegenerative changes in the brains of aged Picciotto *Chrnb2−/−* mutant mice. *Spp1* expression appears to be tissue-dependent in *Chrnb2−/−* mutant mice, but whether it plays a role in neuronal injury response in these animals is not known.

Expression of Lypd2 is markedly downregulated in *Chrnb2−/−* LGNs. LYPD2 is an Ly6 superfamily member closely related to LYNX1, a known modulator of α4ß2 nAChRs [49], [50], [51], [52], [53]. Members of the Ly6 superfamily are present at the tips of growing axons and participate in cell adhesion [52]. LYPD2 is transiently expressed in sensory neurons in the CNS during mouse embryonic development [52]. Morishita et al [53] show that expression of LYNX1 acts as a “molecular brake” by binding to nAChRs in the visual cortex and inhibiting neuronal response at the conclusion of the binocular visual critical period. It is possible that LYPD2 may act in a similar inhibitory manner to focus the segregation of ipsilateral and contralateral axons in the LGN during the retinal wave critical period ending at P8.

CDH1 expression is strongly downregulated and is not detectable in P4 *Chrnb2−/−* mutant LGN. While more detailed analysis is required to address whether genes isolated in the microarray experiments are associated with the reported visual system defects, the finding that CDH1 is specifically down-regulated in the *Chrnb2−/−* mutant LGN is novel and intriguing. CDH1 and CDH2 (N-cadherin) are prototypes of a large family of adhesion molecules that play important roles in the development of the nervous system [54], [55], [56]. Numerous cadherins, including CDH2, are broadly and dynamically expressed in the developing brain and serve diverse functions ranging from control of neuronal morphogenesis to regulation of axonal connections and synapse formation [57]. In contrast, CDH1 is associated with non-neuronal epithelial cells, but expression in the nervous system has been documented [58], [59], [60]. Furthermore, it has been reported that CDH1 is present in chick RGCs and tectum and is required for growth of RGC axons to the tectum [61]. In mice, however, *Cdh1* transcript was not found in embryonic or postnatal retinas tested until P14 [39], consistent with our microarray data for P4.

Our microarray data show expression of C*dh2* mRNA in retina and LGN is high and unaltered in P4 *Chrnb2−/−* mutant mice. It has been established that cadherins, including CDH1 and CDH2, regulate cell-cell interactions through homophilic binding [55]. Neurons and neurites expressing CDH1 or CDH2 are differentially sorted during morphogenesis. Furthermore, it has been reported that CDH1 and CDH2 are present in interneuronal synapses and mediate synaptic adhesion [62]. Because CDH2 is associated with excitatory synapses in cultured hippocampal neurons [63], it has been suggested that CDH1 may be associated with inhibitory synapses [64]. Ablation of CDH1 expression in mutant LGN may interfere with the sorting of axonal projections leading to the defects seen in the *Chrnb2−/−* mutant visual system.

We recognize the limitations of extrapolating from microarray and RT-PCR analysis to protein expression. Nontheless, based on these RNA analyses, it is tempting to hypothesize that deficits of the axon growth regulators CDH1 and LYPD2 are responsible for the diffuse localization of ipsilateral RGC projections in the LGNs of *Chrnb2−/−* mutants. Matter et al [65] proposed that invasion of the chick tectum by RGCs activates expression of CHRNB2. If the same process occurs in the rodent, a mechanism suggests itself: RGCs enter the LGN but without nAChR ß2 subunits, postsynaptic Ca^2+^ activation of transcription is abnormal and expression of CDH1 or LYPD2 in LGN neurons is weakened. The accuracy of neuronal connections in the LGN therefore may be compromised by the altered expression of axon guidance and adhesion molecules at the thalamic level.

## Supporting Information

Figure S1 **Actin controls for RT-PCRs.** Actin amplification controls for RT-PCRs shown in Figures 1–4. Primers are given in Table 1. Ten ng of each prepared RNA template in a 25 µl reaction volume was amplified for 20 cycles. “Control” lanes were amplified without template.
(TIF)

Table S1 **Excel spreadsheets of present calls for all the microarrays.**
(XLS)

Table S2 **Excel spreadsheets of all mutant vs WT comparisons.**
(XLS)

Table S3 **Excel spreadsheets of all Pic vs Xu comparisons.**
(XLS)

Table S4 **Gene Abbreviations and Names from** **Figures 1** **,** **3** **and** **5** **.**
(XLS)
