# Supplementary figures and images for: Mouse Mutants for the Nicotinic Acetylcholine Receptor ß2 Subunit Display Changes in Cell Adhesion and Neurodegeneration Response Genes
Source: PLoS One. 2011 Apr 25;6(4):e18626. doi: 10.1371/journal.pone.0018626 (PMC3081876; doi:10.1371/journal.pone.0018626)

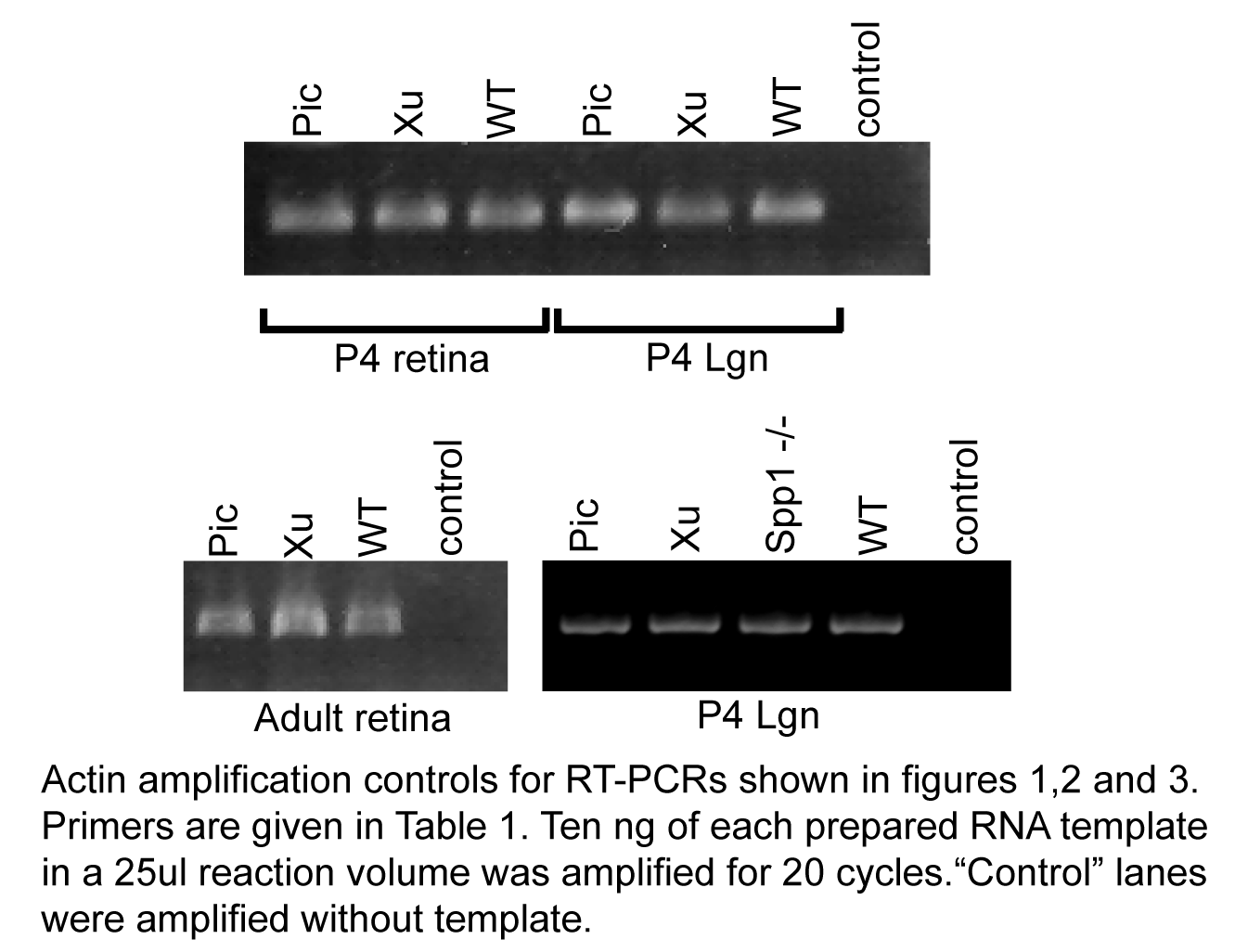

Supplement: Figure S1 — Actin controls for RT-PCRs. Actin amplification controls for RT-PCRs shown in Figures 1– 4. Primers are given in Table 1. Ten ng of each prepared RNA template in a 25 µl reaction volume was amplified for 20 cycles. “Control” lanes were amplified without template. (TIF) [file pone.0018626.s001.tif]
